# Hemipotassium hemirubidium digallium(III) manganese(II) tris(phos­phate) dihydrate

**DOI:** 10.1107/S160053681101258X

**Published:** 2011-04-13

**Authors:** Jing Zhang, Ping Li, Zhi-Hong Liu, Seik Weng Ng

**Affiliations:** a School of Chemistry and Materials Science, Shaanxi Normal University, Xi’an 710062, People’s Republic of China; b Department of Chemistry, University of Malaya, 50603 Kuala Lumpur, Malaysia

## Abstract

The title manganese(II) substituted gallophosphate, K_0.5_Rb_0.5_[Ga_2_Mn(PO_4_)_3_(H_2_O)_2_], features a three-dimensional network built of PO_4_ tetra­hedra, GaO_5_ trigonal bipyramids and MnO_6_ octa­hedra. The Rb^I^ and K^I^ ions, which are disordered with respect to each other in a 1:1 ratio, occupy sites within the channels of the framework. The Rb^I^/K^I^ and Mn^II^ atoms occupy positions of 2 symmetry, as does one of the two P atoms. The Rb^I^/K^I^ site is surrounded by six O atoms [2.996 (2)–3.178 (4) Å] in an irregularly-shaped coordination environment. O—H⋯O hydrogen bonds between the water molecules and phosphate O atoms consolidate the crystal packing.

## Related literature

For isotypic NH_4_[Ga_2_Mn(PO_4_)_3_(H_2_O)_2_], see: Chippindale *et al.* (1998 ▶).

## Experimental

### 

#### Crystal data

K_0.5_Rb_0.5_[Ga_2_Mn(PO_4_)_3_(H_2_O)_2_]
*M* *_r_* = 577.61
Monoclinic, 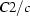

*a* = 13.5504 (12) Å
*b* = 10.2965 (9) Å
*c* = 8.9072 (8) Å
β = 108.527 (1)°
*V* = 1178.34 (18) Å^3^
*Z* = 4
Mo *K*α radiation
μ = 8.31 mm^−1^
*T* = 295 K
0.45 × 0.40 × 0.35 mm

#### Data collection

Bruker SMART APEX diffractometer
Absorption correction: multi-scan (*SADABS*; Sheldrick, 1996 ▶) *T*_min_ = 0.118, *T*_max_ = 0.159
6267 measured reflections
1348 independent reflections
1239 reflections with *I* > 2σ(*I*)
*R*_int_ = 0.037

#### Refinement

*R*[*F* ^2^ > 2σ(*F* ^2^)] = 0.023
*wR*(*F* ^2^) = 0.065
*S* = 1.04
1348 reflections
103 parameters
2 restraints
H-atom parameters constrained
Δρ_max_ = 0.63 e Å^−3^
Δρ_min_ = −0.75 e Å^−3^

### 

Data collection: *APEX2* (Bruker, 2005 ▶); cell refinement: *SAINT* (Bruker, 2005 ▶); data reduction: *SAINT*; program(s) used to solve structure: *SHELXS97* (Sheldrick, 2008 ▶); program(s) used to refine structure: *SHELXL97* (Sheldrick, 2008 ▶); molecular graphics: *X-SEED* (Barbour, 2001 ▶); software used to prepare material for publication: *publCIF* (Westrip, 2010 ▶).

## Supplementary Material

Crystal structure: contains datablocks global, I. DOI: 10.1107/S160053681101258X/br2164sup1.cif

Structure factors: contains datablocks I. DOI: 10.1107/S160053681101258X/br2164Isup2.hkl

Additional supplementary materials:  crystallographic information; 3D view; checkCIF report

## Figures and Tables

**Table 1:** Hydrogen-bond geometry (Å, °)

*D*—H⋯*A*	*D*—H	H⋯*A*	*D*⋯*A*	*D*—H⋯*A*
O1*w*—H1⋯O3^i^	0.84 (3)	1.97 (2)	2.790 (3)	166 (4)
O1*w*—H2⋯O6^ii^	0.84 (3)	2.10 (2)	2.913 (3)	165 (4)

Symmetry codes: (i) 
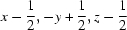
; (ii) 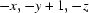.

**Fig. 1.**
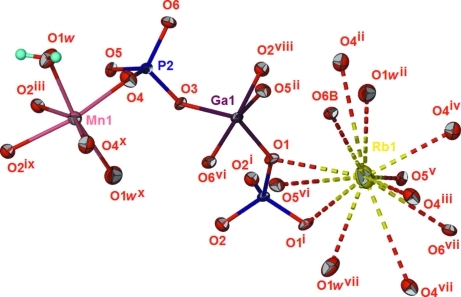
Thermal ellipsoid plot (Barbour, 2001) of a portion of the polymeric structure of K0.5Rb0.5[Ga2Mn(PO4)3(H2O)2] at the 70% probability level; hydrogen atoms are drawn as spheres of arbitrary radius. The potassium atoms are disordered with respect to the rubidium atoms in a 1:1 ratio. Symmetry codes: (i) x + 1/2, -y + 3/2, z + 1/2; (ii) -x + 1/2, -y + 3/2, -z + 1; (iii) -x + 1, y, -z + 3/2; (iv) -x + 1/2, y + 1/2, -z + 1/2; (v) x + 1/2, y + 1/2, z + 1; (vi) x, -y + 1, z + 1/2; (vii) -x + 1, -y + 1, -z + 1; (viii) x, -y + 1, z - 1/2; (ix) -x, y, -z + 1/2; (x) x - 1/2, -y + 1/2, z - 1/2.

## supplementary crystallographic information

### Comment

Microporous aluminium phosphates are readily synthesized by using the hydrothermal route; studies on these compounds have led to improvements in the synthesis of the related gallophosphates. The structure of NH_4_[Ga_2_Mn(PO_4_)_3_(H_2_O)_2_] features PO_4_ tetrahedra, GaO_5_ trigonal bipyramids and MnO_6_ octahedra that are linked together to form a three-dimensional network (Chippindale *et al.*, 1998). The title compound has a similar structure (Fig. 1); however, the rubidium and potassium atoms that occupy the channels within the network rattle in the cavities, as noted from the irregular nature of the polyhedron surrounding the atoms. The coordination number is much higher when longer interactions are considered.

### Experimental

The compound was synthesized from a mixture of gallium oxide (0.037 g), boric acid (0.035 g), rubidium carbonate (0.023 g), potassium carbonate (0.138 g), manganese dichloride tetrahydrate (0.397 g), phosphoric acid (0.15 ml) and water (1.8 ml) (molar ratio of 2:5:1:10:20:20:1000). This mixture was sealed in 25 ml, Teflon-lined, stainless-steel Parr bomb. The bomb was heated at 468 K for 7 days. Colorless block-shaped crystals were isolated.

### Refinement

The water H-atoms were located in a difference Fourier map, and were refined with a distance restraint of O–H 0.84±0.01 Å; their temperature factors were tied to those of the O atom by a factor of 1.5 times.

The potassium and rubidium atoms share the same site, a special position of *2* site symmetry. As the occupancy of each refined to nearly 1/2, the occupancies were then fixed as exactly 1/2. The temperature factors of K1 and Rb1 were restrained to be identical.

### Crystal data

**Table:**

K_0.5_Rb_0.5_[Ga_2_Mn(PO_4_)_3_(H_2_O)_2_]	*F*(000) = 1104
*M**_r_* = 577.61	*D*_x_ = 3.256 Mg m^−^^3^
Monoclinic, *C*2/*c*	Mo *K*α radiation, λ = 0.71073 Å
Hall symbol: -C 2yc	Cell parameters from 4099 reflections
*a* = 13.5504 (12) Å	θ = 2.5–28.6°
*b* = 10.2965 (9) Å	µ = 8.31 mm^−^^1^
*c* = 8.9072 (8) Å	*T* = 295 K
β = 108.527 (1)°	Block, colorless
*V* = 1178.34 (18) Å^3^	0.45 × 0.40 × 0.35 mm
*Z* = 4	

### Data collection

**Table:**

Bruker SMART APEX diffractometer	1348 independent reflections
Radiation source: fine-focus sealed tube	1239 reflections with *I* > 2σ(*I*)
graphite	*R*_int_ = 0.037
ω scans	θ_max_ = 27.5°, θ_min_ = 2.5°
Absorption correction: multi-scan (*SADABS*; Sheldrick, 1996)	*h* = −17→17
*T*_min_ = 0.118, *T*_max_ = 0.159	*k* = −13→13
6267 measured reflections	*l* = −11→11

### Refinement

**Table:**

Refinement on *F*^2^	Primary atom site location: structure-invariant direct methods
Least-squares matrix: full	Secondary atom site location: difference Fourier map
*R*[*F*^2^ > 2σ(*F*^2^)] = 0.023	Hydrogen site location: difference Fourier map
*wR*(*F*^2^) = 0.065	H-atom parameters constrained
*S* = 1.04	*w* = 1/[σ^2^(*F*_o_^2^) + (0.0391*P*)^2^ + 2.8102*P*] where *P* = (*F*_o_^2^ + 2*F*_c_^2^)/3
1348 reflections	(Δ/σ)_max_ = 0.001
103 parameters	Δρ_max_ = 0.63 e Å^−^^3^
2 restraints	Δρ_min_ = −0.75 e Å^−^^3^

### Fractional atomic coordinates and isotropic or equivalent isotropic displacement parameters (Å^2^)

**Table:**

	*x*	*y*	*z*	*U*_iso_*/*U*_eq_	Occ. (<1)
Rb1	0.5000	0.86132 (6)	0.7500	0.03306 (18)	0.50
Ga1	0.32950 (2)	0.57498 (3)	0.42741 (3)	0.01061 (11)	
Mn1	0.0000	0.28198 (6)	0.2500	0.01443 (15)	
K1	0.5000	0.86132 (6)	0.7500	0.03306 (18)	0.50
P1	0.5000	0.49997 (9)	0.7500	0.0106 (2)	
P2	0.21012 (6)	0.37336 (7)	0.17422 (8)	0.01171 (16)	
O1	0.44106 (16)	0.59068 (19)	0.6149 (2)	0.0150 (4)	
O2	0.42851 (16)	0.40472 (19)	0.8014 (2)	0.0154 (4)	
O3	0.29213 (16)	0.41300 (18)	0.3344 (2)	0.0146 (4)	
O4	0.10038 (17)	0.3988 (2)	0.1749 (3)	0.0198 (4)	
O5	0.23416 (16)	0.22845 (19)	0.1603 (2)	0.0156 (4)	
O6	0.22740 (16)	0.45222 (19)	0.0373 (2)	0.0148 (4)	
O1w	−0.11223 (19)	0.3043 (2)	0.0069 (3)	0.0263 (5)	
H1	−0.146 (3)	0.237 (3)	−0.033 (5)	0.039*	
H2	−0.154 (3)	0.366 (3)	−0.001 (5)	0.039*	

### Atomic displacement parameters (Å^2^)

**Table:**

	*U*^11^	*U*^22^	*U*^33^	*U*^12^	*U*^13^	*U*^23^
Rb1	0.0418 (4)	0.0181 (3)	0.0425 (4)	0.000	0.0179 (3)	0.000
Ga1	0.01323 (18)	0.01036 (17)	0.00716 (17)	0.00043 (10)	0.00172 (12)	0.00000 (10)
Mn1	0.0166 (3)	0.0129 (3)	0.0145 (3)	0.000	0.0059 (2)	0.000
K1	0.0418 (4)	0.0181 (3)	0.0425 (4)	0.000	0.0179 (3)	0.000
P1	0.0127 (4)	0.0117 (4)	0.0067 (4)	0.000	0.0021 (3)	0.000
P2	0.0142 (4)	0.0121 (3)	0.0088 (3)	−0.0013 (3)	0.0036 (3)	−0.0006 (2)
O1	0.0181 (10)	0.0163 (10)	0.0072 (9)	−0.0007 (8)	−0.0010 (7)	0.0015 (7)
O2	0.0182 (10)	0.0134 (9)	0.0165 (10)	−0.0009 (8)	0.0081 (8)	0.0005 (8)
O3	0.0213 (11)	0.0110 (9)	0.0101 (9)	−0.0030 (8)	0.0028 (8)	−0.0027 (7)
O4	0.0176 (10)	0.0203 (10)	0.0232 (11)	0.0004 (8)	0.0088 (9)	0.0042 (9)
O5	0.0202 (10)	0.0115 (9)	0.0147 (9)	−0.0038 (8)	0.0048 (8)	−0.0038 (7)
O6	0.0196 (10)	0.0146 (9)	0.0118 (9)	0.0006 (8)	0.0072 (8)	0.0026 (7)
O1w	0.0286 (13)	0.0226 (11)	0.0204 (11)	0.0018 (10)	−0.0025 (9)	−0.0040 (9)

### Geometric parameters (Å, °)

**Table:**

Rb1—O1	3.040 (2)	Mn1—O2^ix^	2.264 (2)
Rb1—O1^i^	3.040 (2)	Mn1—O2^x^	2.264 (2)
Rb1—O4^ii^	3.613 (2)	Mn1—O4	2.079 (2)
Rb1—O4^iii^	2.996 (2)	Mn1—O4^xi^	2.079 (2)
Rb1—O4^iv^	2.996 (2)	Mn1—O1w	2.228 (2)
Rb1—O5^v^	3.555 (2)	Mn1—O1w^xi^	2.228 (2)
Rb1—O5^vi^	3.555 (2)	P1—O1	1.532 (2)
Rb1—O6^vii^	3.451 (2)	P1—O1^i^	1.532 (2)
Rb1—O6^ii^	3.451 (2)	P1—O2	1.546 (2)
Rb1—O4^vii^	3.613 (2)	P1—O2^i^	1.546 (2)
Rb1—O1w^ii^	3.178 (3)	P2—O4	1.512 (2)
Rb1—O1w^vii^	3.178 (3)	P2—O5	1.541 (2)
Ga1—O1	1.870 (2)	P2—O6	1.543 (2)
Ga1—O2^viii^	2.015 (2)	P2—O3	1.558 (2)
Ga1—O3	1.860 (2)	O1w—H1	0.84 (3)
Ga1—O5^ii^	1.850 (2)	O1w—H2	0.84 (3)
Ga1—O6^vi^	1.952 (2)		
			
O4^iv^—Rb1—O4^iii^	68.95 (8)	O4^iii^—Rb1—O4^ii^	95.69 (5)
O4^iv^—Rb1—O1	138.43 (6)	O1—Rb1—O4^ii^	73.69 (5)
O4^iii^—Rb1—O1	139.75 (6)	O1^i^—Rb1—O4^ii^	118.48 (5)
O4^iv^—Rb1—O1^i^	139.75 (6)	O1w^ii^—Rb1—O4^ii^	51.14 (5)
O4^iii^—Rb1—O1^i^	138.43 (6)	O1w^vii^—Rb1—O4^ii^	131.83 (5)
O1—Rb1—O1^i^	47.11 (7)	O6^vii^—Rb1—O4^ii^	134.28 (5)
O4^iv^—Rb1—O1w^ii^	68.68 (6)	O6^ii^—Rb1—O4^ii^	40.88 (5)
O4^iii^—Rb1—O1w^ii^	131.90 (6)	O5^vi^—Rb1—O4^ii^	77.00 (5)
O1—Rb1—O1w^ii^	70.81 (6)	O5^v^—Rb1—O4^ii^	106.30 (5)
O1^i^—Rb1—O1w^ii^	89.44 (6)	O4^iv^—Rb1—O4^vii^	95.69 (5)
O4^iv^—Rb1—O1w^vii^	131.90 (6)	O4^iii^—Rb1—O4^vii^	74.01 (6)
O4^iii^—Rb1—O1w^vii^	68.68 (6)	O1—Rb1—O4^vii^	118.48 (5)
O1—Rb1—O1w^vii^	89.44 (6)	O1^i^—Rb1—O4^vii^	73.69 (5)
O1^i^—Rb1—O1w^vii^	70.81 (6)	O1w^ii^—Rb1—O4^vii^	131.83 (5)
O1w^ii^—Rb1—O1w^vii^	158.73 (9)	O1w^vii^—Rb1—O4^vii^	51.14 (5)
O4^iv^—Rb1—O6^vii^	65.17 (5)	O6^vii^—Rb1—O4^vii^	40.88 (5)
O4^iii^—Rb1—O6^vii^	88.43 (6)	O6^ii^—Rb1—O4^vii^	134.28 (5)
O1—Rb1—O6^vii^	127.06 (5)	O5^vi^—Rb1—O4^vii^	106.30 (5)
O1^i^—Rb1—O6^vii^	83.95 (5)	O5^v^—Rb1—O4^vii^	77.00 (5)
O1w^ii^—Rb1—O6^vii^	93.74 (5)	O4^ii^—Rb1—O4^vii^	167.72 (7)
O1w^vii^—Rb1—O6^vii^	92.00 (5)	O5^ii^—Ga1—O3	123.61 (9)
O4^iv^—Rb1—O6^ii^	88.43 (6)	O5^ii^—Ga1—O1	116.06 (9)
O4^iii^—Rb1—O6^ii^	65.17 (5)	O3—Ga1—O1	120.26 (9)
O1—Rb1—O6^ii^	83.95 (5)	O5^ii^—Ga1—O6^vi^	91.40 (9)
O1^i^—Rb1—O6^ii^	127.06 (5)	O3—Ga1—O6^vi^	87.64 (9)
O1w^ii^—Rb1—O6^ii^	92.00 (5)	O1—Ga1—O6^vi^	93.74 (9)
O1w^vii^—Rb1—O6^ii^	93.74 (5)	O5^ii^—Ga1—O2^viii^	88.85 (8)
O6^vii^—Rb1—O6^ii^	148.53 (7)	O3—Ga1—O2^viii^	88.85 (9)
O4^iv^—Rb1—O5^vi^	131.58 (5)	O1—Ga1—O2^viii^	89.78 (9)
O4^iii^—Rb1—O5^vi^	76.41 (5)	O6^vi^—Ga1—O2^viii^	175.95 (8)
O1—Rb1—O5^vi^	63.43 (5)	O4—Mn1—O4^xi^	109.28 (12)
O1^i^—Rb1—O5^vi^	88.32 (5)	O4—Mn1—O1w	86.67 (9)
O1w^ii^—Rb1—O5^vi^	118.20 (5)	O4^xi^—Mn1—O1w	86.48 (9)
O1w^vii^—Rb1—O5^vi^	55.36 (5)	O4—Mn1—O1w^xi^	86.48 (9)
O6^vii^—Rb1—O5^vi^	147.08 (4)	O4^xi^—Mn1—O1w^xi^	86.67 (9)
O6^ii^—Rb1—O5^vi^	45.70 (4)	O1w—Mn1—O1w^xi^	168.14 (13)
O4^iv^—Rb1—O5^v^	76.41 (5)	O4—Mn1—O2^x^	157.23 (8)
O4^iii^—Rb1—O5^v^	131.58 (6)	O4^xi^—Mn1—O2^x^	93.49 (8)
O1—Rb1—O5^v^	88.32 (5)	O1w—Mn1—O2^x^	94.61 (8)
O1^i^—Rb1—O5^v^	63.43 (5)	O1w^xi^—Mn1—O2^x^	95.46 (8)
O1w^ii^—Rb1—O5^v^	55.36 (5)	O4—Mn1—O2^ix^	93.49 (8)
O1w^vii^—Rb1—O5^v^	118.20 (5)	O4^xi^—Mn1—O2^ix^	157.23 (8)
O6^vii^—Rb1—O5^v^	45.70 (4)	O1w—Mn1—O2^ix^	95.46 (8)
O6^ii^—Rb1—O5^v^	147.08 (4)	O1w^xi^—Mn1—O2^ix^	94.61 (8)
O5^vi^—Rb1—O5^v^	149.86 (6)	O2^x^—Mn1—O2^ix^	63.75 (10)
O4^iv^—Rb1—O4^ii^	74.01 (6)		

Symmetry codes: (i) −*x*+1, *y*, −*z*+3/2; (ii) −*x*+1/2, *y*+1/2, −*z*+1/2; (iii) −*x*+1/2, −*y*+3/2, −*z*+1; (iv) *x*+1/2, −*y*+3/2, *z*+1/2; (v) −*x*+1, −*y*+1, −*z*+1; (vi) *x*, −*y*+1, *z*+1/2; (vii) *x*+1/2, *y*+1/2, *z*+1; (viii) *x*, −*y*+1, *z*−1/2; (ix) −*x*+1/2, −*y*+1/2, −*z*+1; (x) *x*−1/2, −*y*+1/2, *z*−1/2; (xi) −*x*, *y*, −*z*+1/2.

### Hydrogen-bond geometry (Å, °)

**Table:**

*D*—H···*A*	*D*—H	H···*A*	*D*···*A*	*D*—H···*A*
O1w—H1···O3^x^	0.84 (3)	1.97 (2)	2.790 (3)	166 (4)
O1w—H2···O6^xii^	0.84 (3)	2.10 (2)	2.913 (3)	165 (4)

Symmetry codes: (x) *x*−1/2, −*y*+1/2, *z*−1/2; (xii) −*x*, −*y*+1, −*z*.
